# HGD: an integrated homologous gene database across multiple species

**DOI:** 10.1093/nar/gkac970

**Published:** 2022-11-01

**Authors:** Guangya Duan, Gangao Wu, Xiaoning Chen, Dongmei Tian, Zhaohua Li, Yanling Sun, Zhenglin Du, Lili Hao, Shuhui Song, Yuan Gao, Jingfa Xiao, Zhang Zhang, Yiming Bao, Bixia Tang, Wenming Zhao

**Affiliations:** National Genomics Data Center, Beijing Institute of Genomics, Chinese Academy of Sciences and China National Center for Bioinformation, Beijing 100101, China; University of Chinese Academy of Sciences, Beijing 100049, China; National Genomics Data Center, Beijing Institute of Genomics, Chinese Academy of Sciences and China National Center for Bioinformation, Beijing 100101, China; University of Chinese Academy of Sciences, Beijing 100049, China; National Genomics Data Center, Beijing Institute of Genomics, Chinese Academy of Sciences and China National Center for Bioinformation, Beijing 100101, China; University of Chinese Academy of Sciences, Beijing 100049, China; National Genomics Data Center, Beijing Institute of Genomics, Chinese Academy of Sciences and China National Center for Bioinformation, Beijing 100101, China; CAS Key Laboratory of Genome Sciences and Information, Beijing Institute of Genomics, Chinese Academy of Sciences and China National Center for Bioinformation, Beijing 100101, China; National Genomics Data Center, Beijing Institute of Genomics, Chinese Academy of Sciences and China National Center for Bioinformation, Beijing 100101, China; University of Chinese Academy of Sciences, Beijing 100049, China; National Genomics Data Center, Beijing Institute of Genomics, Chinese Academy of Sciences and China National Center for Bioinformation, Beijing 100101, China; CAS Key Laboratory of Genome Sciences and Information, Beijing Institute of Genomics, Chinese Academy of Sciences and China National Center for Bioinformation, Beijing 100101, China; National Genomics Data Center, Beijing Institute of Genomics, Chinese Academy of Sciences and China National Center for Bioinformation, Beijing 100101, China; CAS Key Laboratory of Genome Sciences and Information, Beijing Institute of Genomics, Chinese Academy of Sciences and China National Center for Bioinformation, Beijing 100101, China; National Genomics Data Center, Beijing Institute of Genomics, Chinese Academy of Sciences and China National Center for Bioinformation, Beijing 100101, China; CAS Key Laboratory of Genome Sciences and Information, Beijing Institute of Genomics, Chinese Academy of Sciences and China National Center for Bioinformation, Beijing 100101, China; National Genomics Data Center, Beijing Institute of Genomics, Chinese Academy of Sciences and China National Center for Bioinformation, Beijing 100101, China; CAS Key Laboratory of Genome Sciences and Information, Beijing Institute of Genomics, Chinese Academy of Sciences and China National Center for Bioinformation, Beijing 100101, China; University of Chinese Academy of Sciences, Beijing 100049, China; National Genomics Data Center, Beijing Institute of Genomics, Chinese Academy of Sciences and China National Center for Bioinformation, Beijing 100101, China; CAS Key Laboratory of Genome Sciences and Information, Beijing Institute of Genomics, Chinese Academy of Sciences and China National Center for Bioinformation, Beijing 100101, China; University of Chinese Academy of Sciences, Beijing 100049, China; National Genomics Data Center, Beijing Institute of Genomics, Chinese Academy of Sciences and China National Center for Bioinformation, Beijing 100101, China; CAS Key Laboratory of Genome Sciences and Information, Beijing Institute of Genomics, Chinese Academy of Sciences and China National Center for Bioinformation, Beijing 100101, China; University of Chinese Academy of Sciences, Beijing 100049, China; National Genomics Data Center, Beijing Institute of Genomics, Chinese Academy of Sciences and China National Center for Bioinformation, Beijing 100101, China; CAS Key Laboratory of Genome Sciences and Information, Beijing Institute of Genomics, Chinese Academy of Sciences and China National Center for Bioinformation, Beijing 100101, China; University of Chinese Academy of Sciences, Beijing 100049, China; National Genomics Data Center, Beijing Institute of Genomics, Chinese Academy of Sciences and China National Center for Bioinformation, Beijing 100101, China; CAS Key Laboratory of Genome Sciences and Information, Beijing Institute of Genomics, Chinese Academy of Sciences and China National Center for Bioinformation, Beijing 100101, China; University of Chinese Academy of Sciences, Beijing 100049, China; National Genomics Data Center, Beijing Institute of Genomics, Chinese Academy of Sciences and China National Center for Bioinformation, Beijing 100101, China; CAS Key Laboratory of Genome Sciences and Information, Beijing Institute of Genomics, Chinese Academy of Sciences and China National Center for Bioinformation, Beijing 100101, China; University of Chinese Academy of Sciences, Beijing 100049, China; National Genomics Data Center, Beijing Institute of Genomics, Chinese Academy of Sciences and China National Center for Bioinformation, Beijing 100101, China; CAS Key Laboratory of Genome Sciences and Information, Beijing Institute of Genomics, Chinese Academy of Sciences and China National Center for Bioinformation, Beijing 100101, China; University of Chinese Academy of Sciences, Beijing 100049, China

## Abstract

Homology is fundamental to infer genes’ evolutionary processes and relationships with shared ancestry. Existing homolog gene resources vary in terms of inferring methods, homologous relationship and identifiers, posing inevitable difficulties for choosing and mapping homology results from one to another. Here, we present HGD (Homologous Gene Database, https://ngdc.cncb.ac.cn/hgd), a comprehensive homologs resource integrating multi-species, multi-resources and multi-omics, as a complement to existing resources providing public and one-stop data service. Currently, HGD houses a total of 112 383 644 homologous pairs for 37 species, including 19 animals, 16 plants and 2 microorganisms. Meanwhile, HGD integrates various annotations from public resources, including 16 909 homologs with traits, 276 670 homologs with variants, 398 573 homologs with expression and 536 852 homologs with gene ontology (GO) annotations. HGD provides a wide range of omics gene function annotations to help users gain a deeper understanding of gene function.

## INTRODUCTION

Homology is defined as a common genealogical relationship between and within organisms, which is fundamental to decipher evolutionary processes and infer genes’ potential functions (1–4). Genes with shared ancestry are generally referred to as homologs, which can be further divided into two types, namely, orthologs due to speciation and paralogs due to duplication (5–8). With the advent of comparative genomic research, the identification and inference of homolog relationships relying on sequences have been developed for years, resulting in a large number of practical approaches and databases (9–11), which facilitate the function studies in genomics and systems biology.

In general, the methods of predicting homolog genes mostly based on protein sequence that can be grouped into three categories. The first is graph-based methods that infer gene relationships by calculating pairwise sequences similarity (12), and typically representative databases include COG (13), InParanoid (14), eggNOG (15), Hieranoid (16), OMA (17) and HomoloGene (18). The second is phylogenetic tree-based methods that rely on the reconciliation of gene trees and species trees (19–21), which are adopted by the databases of Panther (22), TreeFam (23) and Ensembl Compara (24). For these two categories, they use a variety of criteria to organize the homologs. For instance, OMA defined grouped homologs, whereas Ensembl Compara used pairwise homologs. The third category is integrated homology prediction methods, which combine multiple algorithms of homology inference, such as DIOPT (25) and Alliance (26). In addition to the methods based on protein sequence prediction, synteny is another method to identify homologs using genomic context (11,27,28), for example, ATGC (29).

The databases mentioned above all provide ongoing maintenance and public data access services except ATGC, but there are still a few issues requiring further improvements. One issue is there is no uniform standard for homology identifier and cross-reference identifier across different databases, resulting in insufficient correlation of identical homologous genes retrieved from different resources. Some databases, for example OMA, used their own defined protein identifier format as homology identifier or used a single cross-reference identifier like Uniprot ID/Ensembl ID/NCBI ID, while others used a combination of Ensembl ID, NCBI gene/protein ID or Uniprot ID as homology identifiers. The divergences in homology identifiers inconvenience the mapping of homologs, which brings barriers to easy access to the most accurate homologs of interest (30). Despite the efforts of the Quest for Orthologs consortium to promote the development of a unified standard for homologs, it remains a challenging task due to the rapid pace of both new genome releases and algorithm updates (30,31). Another issue is that the functional annotations of most homology databases mainly focused on the GO, pathway and/or protein domain, which are not conductive enough to the comprehensive research of homologs based on next-generation sequencing technologies such as evolutionary conservation of gene expression (32). For instance, despite some resources support multi-omics functional annotations like variation and expression, such as Ensembl Compara, they are available in browsing the variation, expression or phenotype information for individual genes but inaccessible to the comparison of homologs across species in a single panel. Additionally, comprehensive homology resources such as DIOPT and Alliance integrates gene symbols, gene identifiers and more functional information about homologs, but focus only on model organisms. Therefore, it is necessary to construct a comprehensive homology resource by integrating multiple homology inference results as well as multi-omics functional annotations and incorporating both model and non-model organisms for the worldwide research communities.

Here, we present the Homologous Gene Database (HGD, https://ngdc.cncb.ac.cn/hgd), a comprehensive homology resource that integrates public homology resources for multi species, incorporates multi-omics gene annotations including traits, variations, gene expression, and gene functional annotations, and provides free public data services for browsing, retrieval, comparison and downloading.

## MATERIALS AND METHODS

### Data source

The inferred homologs, IDs, and gene annotations were collected from worldwide resources. Specifically, HGD integrates predictions from five of the top-performing methods, using the most recent assessment from Quest for ortholog benchmarking (33), including eggNOG (version 5.0, http://eggnog5.embl.de/#/app/home), Panther (version 17.0, http://www.pantherdb.org), TreeFam (version 4.5.1, http://www.treefam.org), Hieranoid (version 2, https://hieranoid.sbc.su.se) and InParanoid (version 8, https://inparanoid.sbc.su.se/cgi-bin/index.cgi). Currently, the inclusion criterion is performing at a higher number of predicted homology relationships and a higher rate of positive predictive values. For ID mapping, a large batch of files with various versions were manually curated and downloaded from Uniprot (https://www.uniprot.org) (34), Ensembl (http://www.ensembl.org) (35) and NCBI (https://www.ncbi.nlm.nih.gov) (36). Gene functional annotations were collected from GWAS Atlas (https://ngdc.cncb.ac.cn/gwas) (37) for traits annotations, GVM (https://ngdc.cncb.ac.cn/gvm) (38) for variants annotations, GEN (https://ngdc.cncb.ac.cn/gen) (39) for expression annotations and Ensembl (http://www.ensembl.org) for GO (40) annotations.

### Data processing

**Figure 1.**
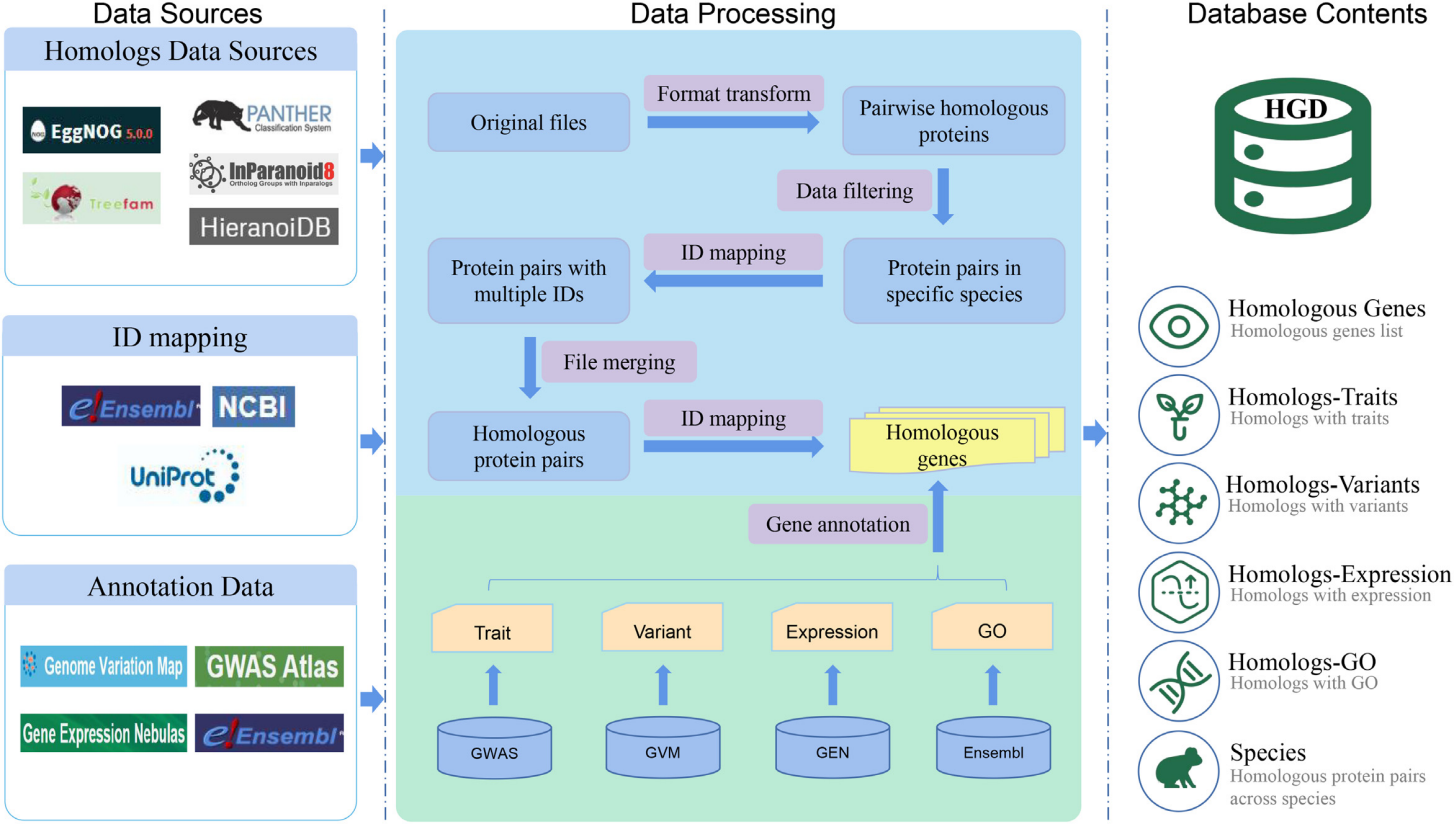
Overview of data sources, data processing and database contents of Homologous Gene Database.

The entire data processing procedure includes homology data pre-process, ID mapping and homologous gene annotation. The collected original homology data was first filtered by 37 species, and then converted into homology pairs. After that, a batch mapping among Ensembl Protein ID, UniProt ID, and NCBI Protein ID was implemented. The basic principle of data integration is that, for each homologous pair compared with others, the conflicting data would be retained and the duplicated data would be merged into a single piece of data. The original homolog ID (e.g. Group ID/ Cluster ID/Tree ID) would be recorded along with the corresponding data sources the homolog comes from in case users need to trace the data. As a result, 112 383 644 non-redundant homologous pairs were obtained. Then, 1 138 192 unique homologous proteins were screened out and complemented with gene basic information including gene identifier, gene symbol, gene synonym, gene type, position, gene description and so on. Subsequently, extensive gene function annotations from GWAS Atlas, GVM, GEN and Ensembl GO were annotated into the above homologous gene list, resulting in homolog annotations for trait, variant, expression and GO respectively. During the data processing, NumPy library and Pandas library of Python with a multi-threaded parallel processing method were used to accelerate the processing of hundreds of millions of homology pairs. The whole process described above is shown in Figure 1.

### Database implementation

HGD was implemented using Spring Boot (https://spring.io/projects/spring-boot; a framework easy to create standalone java applications) as the back-end framework. All data was stored and managed using MySQL (https://dev.mysql.com; a free and popular relational database management system). To provide user-friendly and highly interactive web applications, web pages were constructed using Vue3 (https://v3.cn.vuejs.org/, an approachable, high-performance, and versatile framework for building web user interfaces). Front-end interfaces were built using Element UI (https://element.eleme.cn/; a Vue3 component library for designers and developers). Furthermore, data visualization was built by ECharts (https://www.echarts.com; a JavaScript plug-in for creating interactive charts), D3.js (https://d3js.org/; a JavaScript library for manipulating documents based on data) and DataTables (https://datatables.net; a plug-in for the jQuery JavaScript library to render HTML tables).

## DATABASE CONTENTS AND USAGE

### Homology collection

HGD features comprehensive collection of homology data from diverse resources and integration of multi-omics annotations for multiple species. In the current version, HGD houses 37 species (19 animals,16 plants and 2 microorganisms) with 112,383,644 homologous pairs. Especially, 10 of the 37 species are model organisms. Meanwhile, HGD integrates various annotations from public resources including 16 909 homologs with traits, 276 670 homologs with variants, 398 573 homologs with expression, and 536 852 homologs with GO. The data statistics information is summarized in Table 1.

**Table 1.** Annotation statistics for homologs of each species

Organism	Common name	Taxon Id	#Trait	#Variant	#Expression	#GO
Triticum aestivum	Bread wheat	4565	1102	22 364	25 561	16 602
Manihot esculenta	Cassava	3983	-	-	-	22 121
Gossypium hirsutum	Cotton	3635	-	-	51 141	-
Cucumis sativus	Cucumber	3659	-	-	-	15 636
Phoenix dactylifera	Date palm	42 345	-	-	-	-
Vitis vinifera	Grape	29 760	-	94	-	94
Zea mays	Maize	4577	7 575	-	15 819	26 537
Capsicum annuum	Pepper	4072	-	7 951	-	20 356
Populus trichocarpa	Poplor	3694	-	27 182	-	23 541
Solanum tuberosum	Potato	4113	-	-	-	21 352
Brassica napus	Rapeseed	3708	154	7276	8 466	33 517
Oryza sativa	Rice	4530	6 083	24 796	24 849	20 000
Sorghum bicolor	Sorghum	4558	1 130	26 940	13 776	21 932
Glycine max	Soybean	3847	320	46 583	31 573	38 385
Arabidopsis thaliana	Thale cress	3702	-	-	24 160	22 026
Solanum lycopersicum	Tomato	4081	-	3754	2140	3965
Felis catus	Cat	9685	-	17 764	-	16 686
Bos taurus	Cattle	9913	236	17 738	20 721	17 672
Gallus gallus	Chicken	9031	214	13 327	15 497	13 034
Pan troglodytes	Chimpanzee	9598	-	-	-	19 479
Canis familiaris	Dog	9615	-	-	1077	973
Drosophila melanogaster	Fruit fly	7227	-	-	12 116	10 628
Ailuropoda melanoleuca	Giant panda	9646	-	12 580	-	13 252
Apis mellifera	Honey bee	7460	-	-	-	211
Equus caballus	Horse	9796	-	16 037	-	16 594
Homo sapiens	Human	9606	-	18 969	19 923	19 208
Mus musculus	Mouse	10 090	-	-	21 709	14 495
Sus scrofa	Pig	9823	95	13 315	19 493	11 352
Rattus norvegicus	Rat	10 116	-	-	19 633	17 727
Macaca mulatta	Rhesus monkey	9544	-	-	19 245	16 325
Caenorhabditis elegans	Roundworm	6239	-	-	13 926	10 595
Bombyx mori	Silkworm	7091	-	-	-	7066
Xenopus tropicalis	Tropical clawed frog	8364	-	-	14 064	8427
Meleagris gallapavo	Turkey	9103	-	-	-	10 207
Danio rerio	Zebrafish	7955	-	-	23 415	21 987
Saccharomyces cerevisiae	Brewer's yeast	4932	-	-	269	4295
Escherichia coli	E. coli	562	-	-	-	575

### Homologs with annotated traits

**Figure 2.**
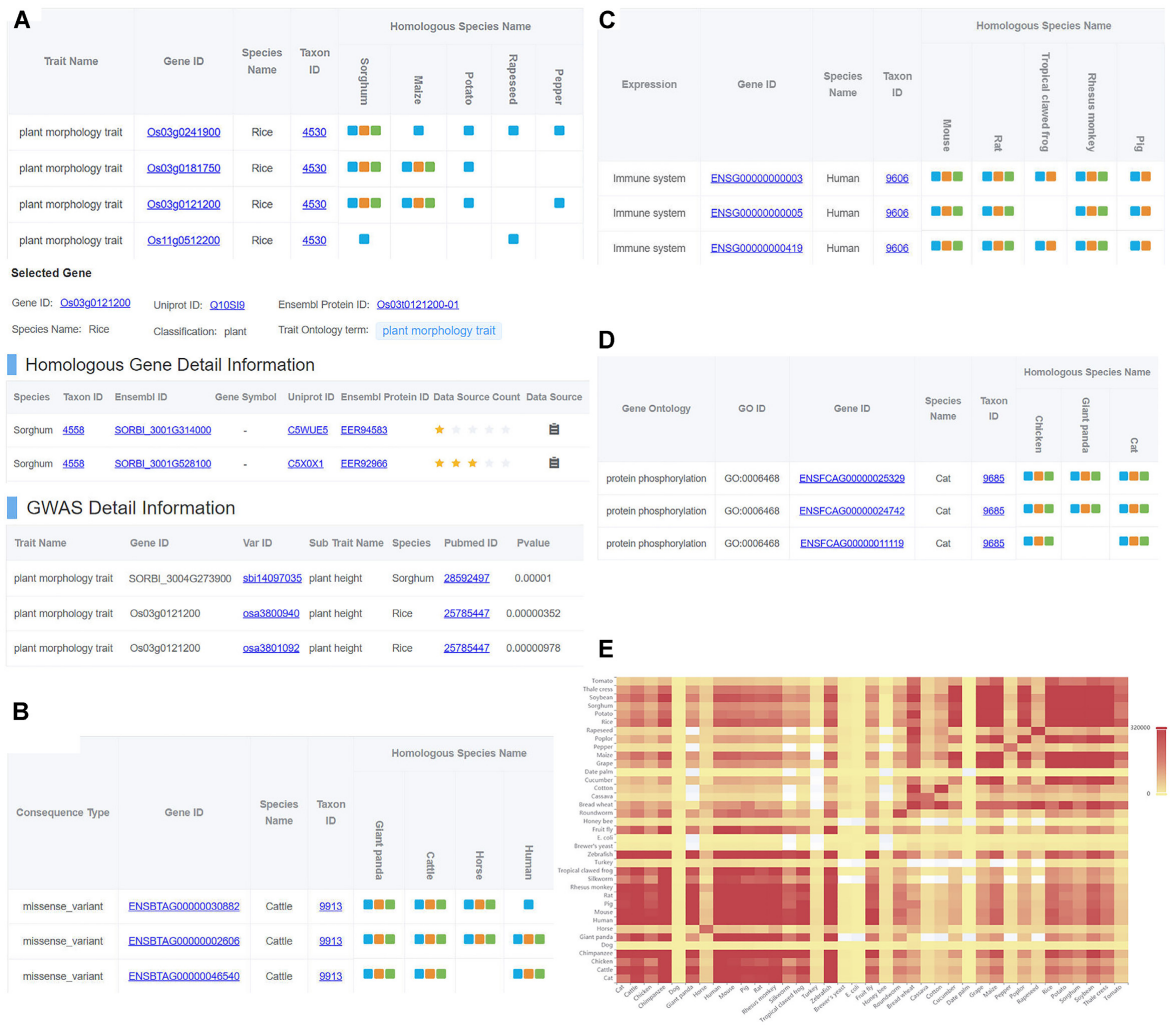
Screenshots of HGD. (**A**) Homology data with trait information and detailed information of homologous genes and GWAS Atlas traits. The blue icon represents homologs between two species, the orange icon represents homologs have ontology term annotations, and the green icon represents two homologs with the same ontology term annotation. (**B**) Homology data with variation information. (**C**) Homology data with expression information. (**D**) Homology data with GO information. (**E**) A heat map of homologous pairs for 37 species.

HGD integrates the trait annotations from GWAS Atlas to provide a more comprehensive understanding of gene function effects on traits (Figure 2A). According to the trait terms in GWAS Atlas, the trait annotations were organized by trait ontology initially obtained from Animal Trait Ontology for livestock (https://bioportal.bioontology.org/ontologies/ATOL) and Plant Trait Ontology (41). After mapping the trait terms to homologous genes, 15 trait ontology terms were filtered with 16 909 homologous genes for 9 species (3 animals and 6 plants). Users can select a trait term of interest to view different trait annotations for multi-species homologs represented by coloured icon. In particular, the green icon indicates the homologs play a same function role in determining given trait. Users can obtain further detailed information by clicking on the green icon, which shows a list of integrated homologs with the number of data sources for quantitative evaluation of the confidence of homologous genes and a list of genotype-phenotype containing detailed genotype information for further research on the gene function of homologs.

### Homologs with associated variants

HGD integrates the variant annotations from GVM and provides the function for comparing various homologous gene variations (Figure 2B). According to the variant annotation results in GVM by Ensembl Variant Effect Predictor (42), the variant annotation data was organized by variant ontology initially selected from the sequence ontology database (http://www.sequenceontology.org) (43). Mapping the variant annotation data to homologous genes resulted in 29 variation terms with 276 670 homologs for 16 species (7 animals and 9 plants). Users can select the variant ontology term of interest to view the distinct variants annotation of multi-species homologs indicated by coloured icon. With the green icons clicked, it presents a homologs list of the clicked gene with detailed information along with a list of variation with detailed allele and positions about that gene, which supports further research on the impact of the variation for homologs.

### Homologs with related expressions

HGD integrates the expression data from GEN to visualize expression profiles of homologs in multiple tissues across species (Figure 2C). According to the expression dataset classification in GEN, the expression data was organized by ontology term initially selected from Disease Ontology (DO, https://disease-ontology.org) (44), BRENDA Tissue Ontology (BTO, http://www.ontobee.org/ontology/bto) and the biological context defined by the GEN. Mapping the expression profiles to homologs resulted in 53 expression terms with 398,573 homologs for 22 species (12 animals, 9 plants and 1 microorganism). When selecting the expression term of interest, a list of the homologs will be displayed. Coloured icons represent the various expression situation of homologs across species. The green icon represents that the homologs may share the same expression pattern, and can be clicked on to further display the homologs list and the average transcripts per million (TPM) value of tissues shown as a boxplot.

### Homologs with annotated GO terms

HGD integrates GO annotation data from Ensembl to provide a functional comparison of genes among homologs (Figure 2D). In GO module, it houses 60 GO terms containing 536,852 homologs of 35 species (19 animals,14 plants and 2 microorganism). Users can select the GO term of interest to view the corresponding homologs across multiple species. A list of integrated homologs and a GO list with detailed sub-GO term are available for further gene function research.

### Homologous pairs between species

HGD provides a heat map of homologous pairs for all 37 species (Figure 2E). From the heat map, it can be observed that there is a large amount of homologous pairs among animals and plants. The two microorganisms have the least number of homologous pairs. Meanwhile, there are a certain number of homologous pairs between animals and plants, which may be valuable for researchers interested in studies across plants and animals. By clicking on the blocks of the heat map, users can directly access a detailed list of homologous genes between any two species.

### Retrieval of homologous genes

HGD provides a basic search function and an advanced data filter function for users to retrieve homologous genes. Users can input various search keywords to search for homologs, including gene symbols, gene synonyms, UniProt ID, Ensembl protein ID, Ensembl gene ID, NCBI gene ID, species common name, protein biotype, gene description and protein name, the latter two with fuzzy matching support. After the search results are obtained, users can further filter using a variety of conditions such as trait ontology, variation ontology, expression term, GO term and species, which can be easily added or removed by a user-friendly web interface. Users can view the gene symbols, Ensembl protein ID and gene description of the resulting homologs. And a homologs list of other species is also available. Meanwhile, users can view the number of gene annotations including GO, expression, variation and trait, and can click for more detailed information. Users can download the results for further data analysis.

### An example of using HGD

**Figure 3.**
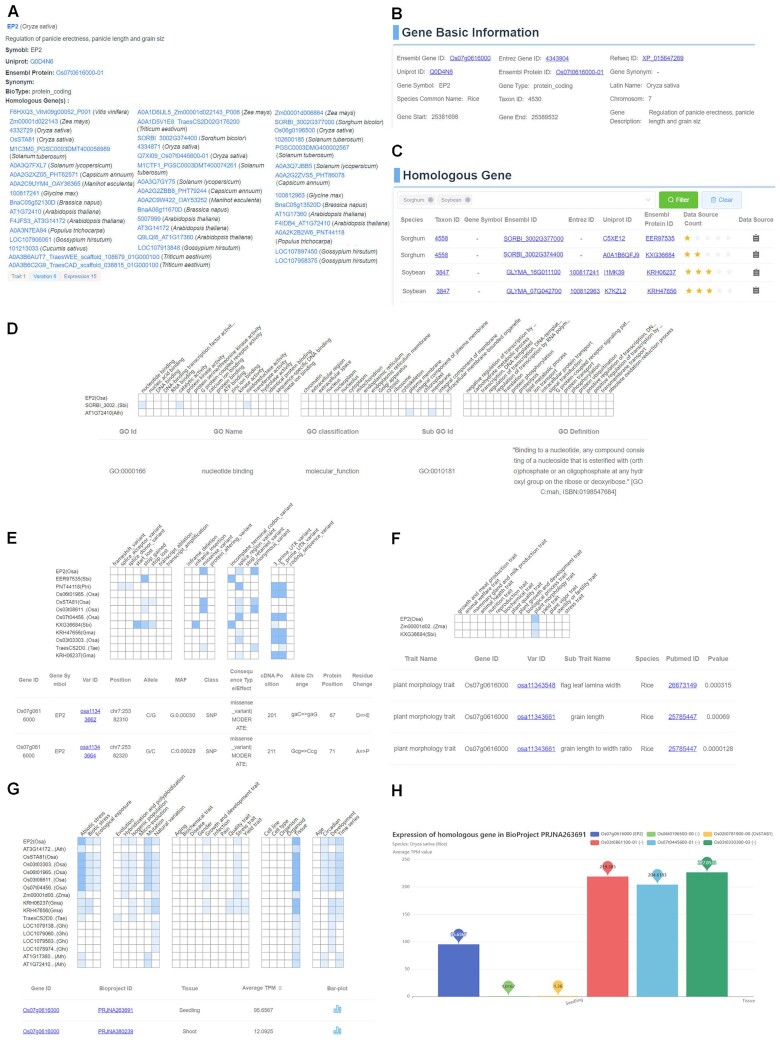
Screenshots of EP2 in HGD. (**A**) A search result for EP2 gene. (**B**) The gene basic information of EP2. (**C**) The homologous gene list of EP2 in rice. (**D**) The GO pattern of EP2 and the homologs, with a list of GO. (**E**) The variation pattern of EP2 and the homologs, with a list of variants. (**F**) The trait pattern of EP2 and the homologs, with a detailed list of GWAS. (**G**) The expression pattern of EP2 and the homologs, with a list of expression values for EP2 in shoot, internode and panicle tissues. (**H**) The bar graph indicating the expression levels of homologous genes in the same RNA-seq dataset.

EP2 is reported to regulate panicle erectness, panicle length, and grain size in rice (45). After searching for EP2 in HGD via gene symbol, the result page shows that EP2 is from Oryza sativa, which has 45 homologous genes across species (Figure 3A). And SORBI_3002G374400 of sorghum being the homologous gene of EP2. Wang *et al.* reported that the EP2 ortholog is a candidate gene for the panicle compactness locus of sorghum and the function needs to be further examined (46). By clicking on EP2 (Oryza sativa), a new page will be open to show 6 sections with gene basic information, homologs, GO, trait, variants and expression. The basic gene information shows gene location, description and various cross-reference IDs of EP2 (Figure 3B). In the homologs section (Figure 3C), all the homology information of EP2, corresponding to 45 homologs, is displayed by default. Filtering the search box for species as sorghum will show that there are two homologous pairs in sorghum. One is Uniprot A0A1B6QFJ9 with family ID PTHR31008 in the homology inference source Panther and multiple IDs such as 3I77Q in the source eggNOG, and the other is C5XE12 with cluster ID 339 in the homology inference source InParanoid (Figure 3C). Each homology inference source comes with a web link, clicking on which will jump to the corresponding homology database. In the GO section, by comparing the homologs, a colored gene function profile normalized by the GO annotation number (Figure 3D) shows that EP2 homologs of sorghum have gene function in nucleotide binding, catalytic activity and oxidoreductase activity. In the variation section, by comparing the homologs, a colored variation profile normalized by the number of variants (Figure 3E) shows that EP2 has missense, splice region and synonymous variants. By clicking on the colored block, a table list is opened to show the detailed variation alleles, positions, molecule consequence, allele change and amino acid residues change, which is useful for further research. In the trait section, a colored trait profile normalized by the number of trait annotation (Figure 3F) shows that both EP2 and the homologs have plant morphology traits. By clicking on the colored block, a GWAS table list is opened to show that EP2 affects flag leaf lamina width (47), grain length and grain length-width ratio (48), whereas the EP2 homologs of sorghum may be associated with panicle morphology (46). In the expression section, by comparing the homologs, a colored expression profile normalized by the number of RNA-seq datasets (Figure 3G) shows *EP2* expressed in a variety of biological contexts including temporal, spatial, phenotypic, genetic and environmental. By clicking on tissue term, the expression data shows that the *EP2* gene is expressed in 31 high-quality RNA-seq datasets and has a high expression level in internode, panicle, embryo (49), shoot (50), coleoptile, root (51), seed, leaf (52,53) and floret, with an average TPM value above 100, which is consistent with the reported high expression of *EP2* in internodes and panicles both temporally and spatially during the heading stage (45). Click on the bar-plot icon and a box graph will pop up to visualize the average TPM values of homologs in the current RNA-seq dataset, which can be used to compare different expression level of homologs in the same RNA-seq dataset (Figure 3H).

## DISCUSSION AND FUTURE PLANS

Homologs are genes with shared ancestry (5), which plays a crucial role for comparative, developmental, and molecular biology. Homolog database as the curated knowledgebase also plays an important role in genome-related research, and there are already lots of homologous gene databases released (13–18,22–26,29). Different from these databases (Table 2), HGD systematically integrate homologs from 5 public single homologs resources including eggNOG, Panther, TreeFam, Hieranoid and InParanoid, and with some specific features. HGD uses a homologous gene naming rule to display homologs primarily in gene symbols. By handling a wide range of ID mappings, HGD supports searching for homologs by various keywords, including gene symbol, gene synonym, protein name, Uniprot ID, Ensembl protein ID, Ensembl gene ID, NCBI gene ID and so on. Meanwhile, HGD collects multi-omics data including trait, expression and variation from 3 public resources including GWAS Atlas (37), GVM (38) and GEN (39) of NGDC (54) and provides a comparison function when browsing homologs of multiple species simultaneously, together with search functions by genes, species and ontology terms to facilitate convenient access to data of interest. In addition, HGD houses a number of species including animals, plants and microorganisms, which helps to extend the homologous genes research to non-model organisms. Since HGD has integrated homologous genes with multi-omics annotation data, users can explore the functional effects of homologous genes on traits from different species, compare the variety of homologous gene variations and demonstrate the difference in expression levels of homologs in multiple tissues across species. All these features set HGD apart from all existing homology resources, and make HGD, as a complement to existing resources, an indispensable and important homologous gene resource in the community.

**Table 2.** The overall features of existed homology resources

					Gene annotation types						Homologs view mode		
Database	Homology inferring method	Homology relationship	#Organisms/ species	Homologs’ ID type	GO	Pathway	Protein/ domain ID	Trait	Variation	Expression	Single gene	By sequence	By comparing
COG	Graph-based	Pairwise, group	1309	NCBI Protein ID	×	√	√	×	×	×	√	×	×
InParanoid	Graph-based	Group	273	Uniprot ID	×	×	√	×	×	×	√	√	×
eggNOG	Graph-based	Group	5090	NCBI/Ensembl/ Uniprot Protein ID	√	√	√	×	×	×	√	√	×
OMA	Graph-based	Pairwise, group	2326	Self-definded Protein ID, Uniprot ID/ Ensembl ID/ NCBI ID	√	×	√	×	×	×	√	√	×
HomoloGene	Graph-based	Group	21	Gene symbol/NCBI Gene ID	×	×	√	√	√	√	√	√	×
Panther	Tree-based	Group	-	Ensembl/NCBI Gene ID,Protein ID	√	√	×	×	×	×	√	√	×
Hieranoid	Tree-based	Group	66	Uniprot ID	×	×	×	×	×	×	√	√	×
TreeFam	Tree-based	Pairwise, group	109	Ensembl Gene ID/Gene Name/ Uniprot ID	×	×	√	×	×	×	√	√	×
Ensembl Compara	Tree-based	Pairwise	-	Ensembl Gene ID	√	√	×	√	√	√	√	√	×
DIOPT	Integrated	Pairwise	10^a^	Ensembl/NCBI Gene ID	√	×	×	√	×	×	√	×	×
Alliance	Integrated	Pairwise	7^a^	Gene symbol	√	√	×	√	√	√	√	×	√

^a^ Mainly for model organisms.

In the future, we plan to continuously update and integrate homologs from high-quality resources such as OMA (17) and OrthoDB (55) to enlarge the homology resource and curate reported or validated homologs from public papers to provide more high-confidence homologous relationships. Meanwhile, we will add more organisms, such as cultivars like Sweet potato, Rye and Green gram to fulfil various research requirements. In addition, we will develop online tools such as homology visualization and BLAST (56) to help users retrieve and browse homologs with annotated data in a more user-friendly manner.

## DATA AVAILABILITY

HGD is available online for free at https://ngdc.cncb.ac.cn/hgd and does not require user registration.
